# Insights into the evolutionary history of Japanese encephalitis virus (JEV) based on whole-genome sequences comprising the five genotypes

**DOI:** 10.1186/s12985-015-0270-z

**Published:** 2015-03-14

**Authors:** Xiaoyan Gao, Hong Liu, Minghua Li, Shihong Fu, Guodong Liang

**Affiliations:** State Key Laboratory for Infectious Disease Prevention and Control, National Institute for Viral Disease Control and Prevention, Chinese Center for Disease Control and Prevention, Beijing, 102206 China; Collaborative Innovation Center for Diagnosis and Treatment of Infectious Diseases, Hangzhou, 310003 China; School of Life Sciences, Shandong University of Technology, Zibo, Shandong China

**Keywords:** Japanese encephalitis virus, Genotype, Genetic diversity

## Abstract

**Background:**

Japanese encephalitis virus (JEV) is the etiological agent of Japanese encephalitis (JE), one of the most serious viral encephalitis worldwide. Five genotypes have been classified based on phylogenetic analysis of the viral envelope gene or the complete genome. Previous studies based on four genotypes have reported that in evolutionary terms, genotype 1 JEV is the most recent lineage. However, until now, no systematic phylogenetic analysis was reported based on whole genomic sequence of all five JEV genotypes.

**Findings:**

In this study, phylogenetic analysis using Bayesian Markov chain Monte Carlo simulations was conducted on the whole genomic sequences of all five genotypes of JEV. The results showed that the most recent common ancestor (TMRCA) for JEV is estimated to have occurred 3255 years ago (95% highest posterior density [HPD], −978 to−6125 years). Chronologically, this ancestral lineage diverged to produce five recognized virus genotypes in the sequence 5, 4, 3, 2 and 1. Population dynamics analysis indicated that the genetic diversity of the virus peaked during the following two periods: 1930–1960 and 1980–1990, and the population diversity of JEV remained relatively high after 2000.

**Conclusions:**

Genotype 5 is the earliest recognized JEV lineage, and the genetic diversity of JEV has remained high since 2000.

## Findings

Japanese encephalitis virus (JEV) is the prototype member of the JEV serogroup within the genus Flavivirus, family *Flaviviridae*. JEV comprises five genotypes (G1-G5) [1-3]. In previous studies, the phylogenetic characteristics of JEV were analyzed and the most recent common ancestor (TMRCA) was estimated. The TMRCA of JEV was estimated to be 1690 years when calculations were based on the complete sequence of four genotypes (G1-G4) [4], whereas, analysis of JEV using a limited number of whole genomic sequences from five genotypes indicated that TMRCA of JEV appeared approximately 460 years [5]. More recently, however, G5 strain XZ0934 isolated in 2009, which had not been included in earlier analyses, was shown to be significantly different from the G5 Muar isolate [6]. Therefore, in order to improve our understanding of the evolutionary progress and population diversity of JEV, a comprehensive dataset was established for evolutionary analysis of JEV in this study. In the dataset, 100 whole genomic sequences of JEV representing all five genotypes of JEV, isolated from various hosts (humans, pigs and bats) and vectors (mosquitoes and midges) were collected and analyzed.

Two G5 JEV full-length genome sequences (Muar and XZ0934) were downloaded from GenBank (GB No. HM596272 and JF915894, respectively) and added to the database established in a previous report [4], forming a new database for analysis (Table 1). The new JEV sequence database was analyzed using Bayesian Markov chain Monte Carlo (MCMC) method. The General Time Reversible (GTR) model + Invariant (I) + Gamma (G) model was selected using MrModelTest [7]. The nucleotide substitution rates and divergence times of the most recent common ancestor (TMRCA) were estimated using the relaxed (uncorrelated lognormal) molecular clock model in the BEAST software package [8]. Demographic histories of JEV were inferred based on Bayesian skyline reconstruction. The analysis was run through 1,000,000,000 generations to ensure sufficient mixing. Finally, the maximum clade credibility (MCC) tree was built using TreeAnnotator with 10% burn-in (http://beast.bio.ed.ac.uk/).

**Table 1:** **Information of JEV isolates analyzed in this study**

**Strain**	**Date**	**Country**	**Host** ^**a**^	**Genotype**	**GenBank accession no.**
47	1950’s	China:Heilongjiang	CSF	3	JF706269
14178	2001	India	-	3	EF623987
57434	2005	India	-	3	EF623988
04940-4	2002	India	-	3	EF623989
B58	1989	China:Yunnan	Bat	3	FJ185036
Beijing-1	1949	China	Human brain	3	L48961
BL06-50	2006	China:Guangxi	*Culex tritaeniorhynchus*	1	JF706270
BL06-54	2006	China:Guangxi	*Culex tritaeniorhynchus*	1	JF706271
CBH	1954	China:Fujian	CSF	3	JN381860
CH-13	1957	China:Sichuan	CSF	3	JN381870
CH1392	1990	Taiwan	*Culex tritaeniorhynchus*	3	AF254452
CTS	1955	China:Fujian	CSF	3	GQ429184
CZX	1954	China:Fujian	CSF	3	JN381865
DH107	1989	China:Yunnan	*Aedes lineatopennis*	3	JN381873
DL04-29	2004	China:Yunnan	Culex theileri	3	JF706272
DL04-45	2004	China:Yunnan	*Ar. Subalbatus & Mansonia uniform*	3	JN381854
Fj02-29	2002	China:Fujian	CSF	3	JF706273
Fj02-76	2002	China:Fujian	Human blood	3	JN381867
FJ03-39	2003	China:Fujian	Human blood	3	JN381859
FJ03-94	2003	China:Fujian	Human blood	3	JN381858
FU	1995	Australia	Human serum	2	AF217620
G35	1954	China:Fujian	Mosquito pool	3	GQ429185
GB30	1997	China:Yunnan	*Murina aurata brain tissue*	3	FJ185037
GP78	1978	India	Human brain	3	AF075723
GS07-TS11	2007	China:Gansu	*Culex tritaeniorhynchus*	1	JN381843
GSBY0801	2008	China:Gansu	*Culex tritaeniorhynchus*	1	JF706274
GSBY0804	2008	China:Gansu	*Culex tritaeniorhynchus*	1	JN381844
GSBY0810	2008	China:Gansu	*Culex tritaeniorhynchus*	1	JN381840
GSBY0816	2008	China:Gansu	*Culex tritaeniorhynchus*	1	JN381842
GSBY0827	2008	China:Gansu	*Culex tritaeniorhynchus*	1	JN381845
GSBY0861	2008	China:Gansu	*Culex tritaeniorhynchus*	1	JN381833
GSS	1960’s	China:Beijing	CSF	3	JF706275
GX0519	2005	China:Guanxi	*Culex tritaeniorhynchus*	1	JN381835
GX0523/44	2005	China:Guanxi	*Culex tritaeniorhynchus*	1	JN381832
GZ04-2	2004	China:Guizhou	*Armigeres*	3	JN381857
GZ56	2006	China:GuiZhou	CSF	1	HM366552
Ha-3	1960’s	China:Heilongjiang	CSF	3	JN381872
HB49	1990	China:Yunnan	*Rousettus leschenaulti* blood	3	JF706284
HB97	1990	China:Yunnan	*Rousettus leschenaulti* blood	3	JF706285
HLJ02-134	2002	China:Heilongjiang	*Genus culicoides*	3	JF706276
HN04-11	2004	China:Henan	Culex	1	JN381831
HN04-21	2004	China:Henan	Culex	1	JN381841
HN06129	2006	China:Henan	*Armigeres*	1	JF706277
HN0621	2006	China:Henan	Culex	1	JN381830
HN0626	2006	China:Henan	Culex	1	JN381837
HVI	1965	Taiwan	Mosquito	3	AF098735
HYZ	1979	China:Yunnan	Patient blood	3	JN381853
Ishikawa	1994	Japan	*Culex tritaeniorhynchus*	1	AB051292
JaGAr 01	1959	Japan	*Cluex*	3	AF069076
JaOArS982	1982	Japan	Mosquito	3	M18370
JaOH0566/Japan/1966/human	1966	Japan	Human	3	AY508813
JEV/sw/Mie/40/2004	2004	Japan	Swine serum	1	AB241118
JEV/sw/Mie/41/2002	2002	Japan	Swine serum	1	AB241119
JH04-18	2004	China:Yunnan	*Whitmorei & Anophelessinensis*	3	JN381855
JKT6468	1981	Indonesia	Mosquito	4	AY184212
K87P39	1987	South Korea	Mosquito	3	AY585242
KV1899	1999	Korea	Swine	1	AY316157
LFM	1955	China:Fujian	Human blood	3	JN381863
Ling	1965	Taiwan	Human brain	3	L78128
LN02-102	2002	China:liaoning	*Culex modestus*	1	JF706278
LN0716	2007	China:Liaoning	*Culex tritaeniorhynchus*	1	JN381849
LYZ	1957	China:Fujian	CSF	3	JN381869
M28	1977	China:Yunnan	*Culex pseudovishnui*	1	JF706279
Nakayama	1935	Japan	Human brain	3	EF571853
P3	1949	China:Beijing	Human brain	3	U47032
RP-2 ms	1985	Taiwan	Mosquito	3	AF014160
RP-9	1985	Taiwan	Mosquito	3	AF014161
SA14	1954	China	Mosquito	3	U14163
SC04-12	2004	China:Sichuan	*Culex*	1	JN381839
SC04-15	2004	China:Sichuan	*Culex tritaeniorhynchus*	1	JN381838
SD0810	2008	China:Shandong	*Culex tritaeniorhynchus*	1	JF706286
SH03-103	2003	China:Shanghai	*Culex tritaeniorhynchus*	1	JN381847
SH03-105	2003	China:Shanghai	*Culex tritaeniorhynchus*	1	JN381846
SH04-10	2004	China:Shanghai	*Culex tritaeniorhynchus*	3	JN381856
SH04-5	2004	China:Shanghai	*Culex tritaeniorhynchus*	3	JN381866
SH17M-07	2007	China	-	1	EU429297
SH-3	1987	China:Shanghai	CSF	3	JN381864
SH-53	2001	China:Shanghai	*Culex tritaeniorhynchus*	1	JN381850
SH-80	2001	China:Shanghai	*Culex tritaeniorhynchus*	1	JN381848
T1P1	1997	Taiwan	*Armigeres subalbatus*	3	AF254453
TLA	1971	China:Liaoning	CSF	3	JN381868
Vellore P20778	1958	India	Human brain	3	AF080251
XJ69	2007	China	*Culex pipiens pallens*	1	EU880214
XJP613	2007	China	*Culex tritaeniorhynchus*	1	EU693899
XZ0938	2009	China:Xizang	*Culex tritaeniorhynchus*	1	HQ652538
YLG	1955	China:Fujian	CSF	3	JF706280
YN	1954	China:Yunnan	CSF	3	JN381871
YN05124	2005	China:Yunnan	*Culex tritaeniorhynchus*	1	JF706281
YN05155	2005	China:Yunnan	*Culex tritaeniorhynchus*	1	JN381852
YN0623	2006	China:Yunnan	*Culex tritaeniorhynchus*	1	JN381836
YN0911	2009	China:Yunnan	*Culex tritaeniorhynchus*	1	JF706267
YN0967	2009	China:Yunnan	*Culex tritaeniorhynchus*	1	JF706268
YN79-Bao83	1979	China:Yunnan	*Culex tritaeniorhynchus*	1	JN381851
YN82-BN8219	1982	China:Yunnan	Mosquito	1	JN381834
YN83-Meng83-54	1983	China:Yunnan	*Lasiohelea taiwana Shiraki*	1	JF706282
YN98-A151	2003	China:Yunnan	Mosquitoes	3	JN381861
ZMT	1955	China:Fujian	CSF	3	JF706283
ZSZ	1955	China:Fujian	CSF	3	JN381862
Muar	1952	Malaysia	Human brain	5	HM596272
XZ0934	2009	China:Tibet	*Culex tritaeniorhynchus*	5	JF915894

^a^- Information not available.

**Figure 1:**
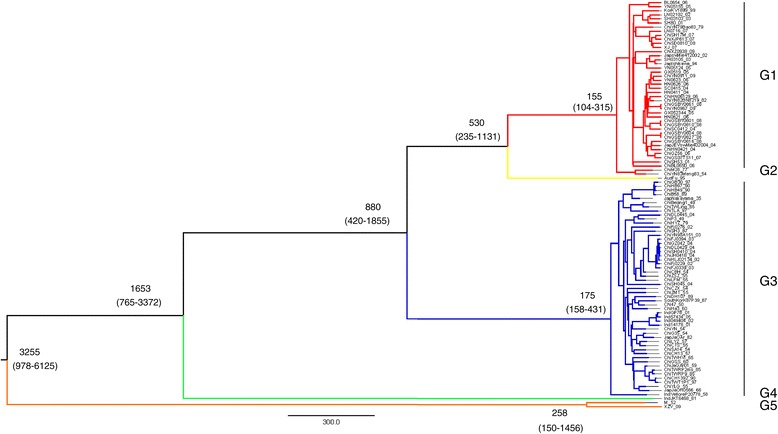
**Maximum clade credibility (MCC) tree for 100 whole-genome sequences of JEV.** Five distinct lineages were identified: G1 (red), G2 (yellow), G3 (blue), G4 (green) and G5 (orange). Estimated TMRCAs of these lineages (with their 95% HPD values in parentheses) are shown, G1: 155(104–315), G2: 530(235–1131), G3: 880(420–1855), G4: 1653(765–3372), and G5: 3255(978–6125).

Based on Bayesian Markov chain Monte Carlo (MCMC) analysis, the maximum clade credibility (MCC) tree for the whole genomic sequences of JEV was established (Figure 1). Representatives of the five distinct lineages were included in the analysis. The posterior probability values for the nodes of each lineage were >0.95, indicating their robustness. JEV was estimated to have emerged 3255 years ago (95% HPD: −978 to −6125 years) and subsequently diverged at least five times to produce the 5 recognized genotypes. In chronological order, they diverged in the order G5, G4, G3, G2 and G1. Thus, G5 represents the most ancestral lineage among genotypes 1–5.

The mean rate of nucleotide substitution for the whole genomic sequences of 100 JEV strains isolated from a variety of hosts worldwide, estimated using a Bayesian MCMC approach, was 1.01 × 10^−4^ nucleotide substitutions per site per year (95% HPD values, 4.37 × 10^−5^, 1.56 × 10^−4^). This is similar to previous estimates based on analysis of four JEV genotypes [4].

**Figure 2:**
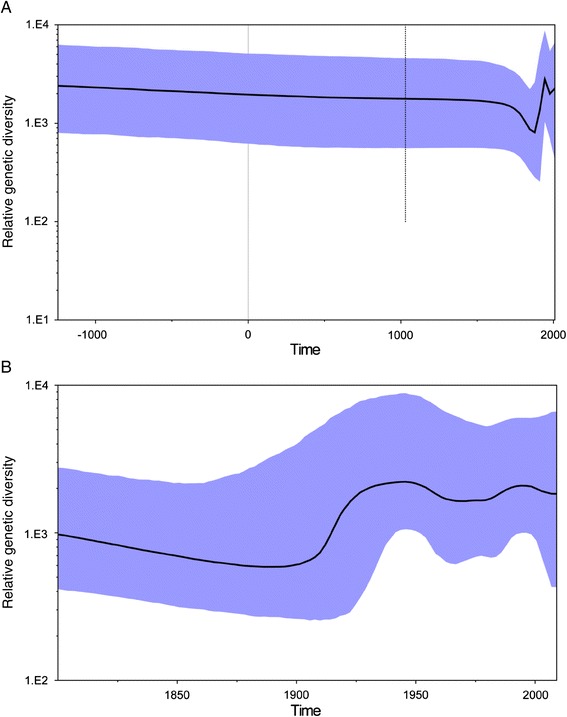
**Bayesian skyline plots for JEV.** Highlighted areas correspond to 95% HPD intervals. **(A)** Populations during the whole evolutionary history; **(B)** Populations during the later evolutionary history since 1800.

The population dynamics of JEV are shown in Figure 2. The skyline plot showed that the JEV population had experienced complicated changes during the process of evolution. However, the virus population remained relatively stable during the first 2700 years (Figure 2A), followed by a period of rapid decline from the 1700s, reaching a minimum in the 1900s. It then increased rapidly from the 1930s until the 1960s and formed the first peak. The second peak appeared in the 1980-1990s and subsequently the populations of JEV remained high after 2000 (Figure 2B).

The findings in this study have similarities with previous studies [5]. For example, the divergence pattern of the genotypes occurred in the order G5, G4, G3, G2 and G1, and the mean rate of nucleotide substitution was similar to previous estimates. However, the occurrence time of TMRCA determined in this study (~3255 years ago) was quite different compared with that measured in the report (~460 years ago) of Mohammed et al. [5]. The reason for this discrepancy could be attributed to the dataset used for analysis. In Mohammed’s study, only 35 whole genomic sequences were used and the only G5 representative included was the Muar strain. Therefore, since our new dataset includes two G5 representatives with robust sequences, the occurrence time of TMRCA (~3255 years ago) obtained in this study should reflect more precisely the evolutionary patterns and diversity of JEV.

Two main peak periods in population dynamics were identified in this study, 1930–1960 and 1980-1990s, respectively. These fluctuations were reflections of the virus activity in the sylvatic environment. Since the 1930s, JEV strains belonging to G3 emerged and were isolated from Asian countries. G5 was first identified in 1952 [2,4]. This was a good interpretation of the first peak. Subsequently, G2 and G4 strains were isolated during the 1980s. Importantly, the G1 genotype emerged during the 1980s onwards [4]. Therefore, the virus population diversity peaked in the 1980-1990s. Since 2000, G1 JEV has become the dominant genotype in most endemic regions [4], and although a relatively small decrease was observed, the virus remains the most active and G5 reemerged.

Interestingly, although G5 is estimated to be the most ancestral JEV lineage, this virus showed a highly active dispersal capacity following its reemergence. Indeed, this new G5 strain was isolated from mosquitoes collected in southern region of the Asian continent (Tibet, China) in 2009 [2] and northeast region of Asia (South Korea) during the same year [9]. Thus, G5 now appears to be dispersing widely in Asia. A recent study showed that genotype 1 JEV originated in Southeastern Asia and spread to the entire Asian continent [10]. Based on these observations, it seems likely that G5 will follow a dispersal pattern similar to that of G1 JEV, and has dispersed or will disperse over the entire Asian continent. Clearly, G5 should be monitored closely throughout JEV endemic regions.

Finally, the available inactivated and live attenuated JE vaccines are derived from G3 JEVs [11]. Thus, the level of cross protection of the current vaccines against G5 JEV is likely to be sub-optimal and should therefore be analyzed carefully since the reemergence of G5 and its widespread dispersal, and significant genetic variation could impact on its epidemiology. This possibility is emphasized by the fact that Muar (the first G5 JEV) strain was isolated from a patient with severe viral encephalitis [12]. Thus, there is the realistic possibility that the newly isolated G5 viruses could be highly virulent. Thus, the potential disease burden of viral encephalitis caused by G5 JEV requires careful reassessment.

## Abbreviations

JEV: Japanese encephalitis virus
JE: Japanese encephalitis
E: Envelope
TMRCA: The most recent common ancestor
HPD: Highest posterior density
G: Genotype
MCMC: Markov chain Monte Carlo
MCC: Maximum clade credibility
Ser: Serine
ORF: Open reading frame
